# Probabilistic Hesitant Fuzzy Evidence Theory and Its Application in Capability Evaluation of a Satellite Communication System

**DOI:** 10.3390/e26010094

**Published:** 2024-01-22

**Authors:** Jiahuan Liu, Ping Jian, Desheng Liu, Wei Xiong

**Affiliations:** Science and Technology on Complex Electronic System Simulation Laboratory, Space Engineering University, Beijing 101400, China; jianping85730@sina.com (P.J.); liudsnudt@126.com (D.L.); 13331094335@163.com (W.X.)

**Keywords:** Dempster–Shafer evidence theory, probabilistic hesitant fuzzy set, capability evaluation, satellite communication system

## Abstract

Evaluating the capabilities of a satellite communication system (SCS) is challenging due to its complexity and ambiguity. It is difficult to accurately analyze uncertain situations, making it difficult for experts to determine appropriate evaluation values. To address this problem, this paper proposes an innovative approach by extending the Dempster-Shafer evidence theory (DST) to the probabilistic hesitant fuzzy evidence theory (PHFET). The proposed approach introduces the concept of probabilistic hesitant fuzzy basic probability assignment (PHFBPA) to measure the degree of support for propositions, along with a combination rule and decision approach. Two methods are developed to generate PHFBPA based on multi-classifier and distance techniques, respectively. In order to improve the consistency of evidence, discounting factors are proposed using an entropy measure and the Jousselme distance of PHFBPA. In addition, a model for evaluating the degree of satisfaction of SCS capability requirements based on PHFET is presented. Experimental classification and evaluation of SCS capability requirements are performed to demonstrate the effectiveness and stability of the PHFET method. By employing the DST framework and probabilistic hesitant fuzzy sets, PHFET provides a compelling solution for handling ambiguous data in multi-source information fusion, thereby improving the evaluation of SCS capabilities.

## 1. Introduction

The satellite communication system (SCS) consists of communications satellites, tracking and data relay satellites, and associated ground stations that provide data transmission to support ground systems. It demands various functions, performance, and effectiveness to carry out complex and diverse mission tasks successfully. Analyzing the capabilities of SCS is crucial to ensure its reliability and performance in meeting expectations. In addition, the primary objective of evaluating the capability satisfaction of the SCS is to identify vulnerabilities and performance bottlenecks and establish a foundation for future technology and system upgrades. Therefore, finding an accurate method to evaluate the capability of SCS is crucial.

Numerous researchers have used various techniques to evaluate the efficiency and performance of SCS, such as mathematical modeling [1], Bayesian theory [2], gray integrated evaluation [3], ADC method [4], neural network [5], and intuitionistic fuzzy set (IFS) [6]. However, there are still relatively few mature methods available for evaluating the satisfaction degree of SCS capability requirements.

The Dempster–Shafer evidence theory (DST) [7] is a probabilistic reasoning method for multi-source information fusion with uncertainty, ambiguity, and incompleteness. It has been extensively studied in terms of evidential reasoning [8,9], belief entropy [10,11], uncertainty measure [12], belief divergence measure [13], and other hybrid approaches [14,15,16].DST is widely applied in various fields, including classification [17,18,19], decision making [20], risk assessment [21,22], medical diagnosis [23], and others, and others, as it offers rigorous and effective data fusion solutions.

In traditional DST, the probability assignment of a focal element represents the level of support for a proposition and is typically a precise value. However, in situations where evaluation criteria are ambiguous and experts are hesitant to express their opinions, the probability assignment may become uncertain or inaccurate. To address this challenge, fuzzy sets such as IFS have been employed for decision-making under uncertainty [24]. As a result, researchers have integrated DST with various approaches such as IFS, hesitant fuzzy linguistic term sets, Pythagorean fuzzy sets, hesitant fuzzy sets (HFS) theory, and others to capture and describe uncertainty in the decision-making process [20,25,26,27].

HFS was developed by Torra and Narukawato [28,29] as an extension of fuzzy sets that models the hesitancy by allowing multiple degrees of membership values for each element. It has been proven that the envelope of the HFSs is an IFS [28]. Compared to IFS, HFS provides more precise information descriptions, enabling accurate characterization of the degree associated with each evaluation value. Moreover, HFS aligns with people’s intuitive feelings and diversity of opinions. Therefore, HFS is a valuable tool for decision-making and analysis, especially in uncertain and ambiguous scenarios.

However, all elements in the hesitant fuzzy element (HFE) have equal occurring probabilities, implying equal importance for all membership values. To address this issue, Xu and Zhou [30] introduced the probabilistic hesitant fuzzy set (PHFS) and probabilistic hesitant fuzzy element (PHFE), which generalize the fuzzy form by incorporating probabilities with the associated membership values. The academic community has shown considerable interest in the research on PHFS, leading to numerous findings [31,32] since PHFS enables more precise characterization of fuzziness and hesitation in human thinking. In [33], a ranking model under PHFS is introduced by extending the concept of evidence theory. However, this approach converts probabilistic hesitant fuzzy information into a single value before combining it with evidence theory. This premature transformation eliminates uncertainty in PHFS, potentially overlooking uncertainties and hesitations important for decision-making in complex systems.

Therefore, in this paper, we extend the evidence theory to the probabilistic hesitant fuzzy evidence theory (PHFET). The probabilistic hesitant fuzzy basic probability assignment (PHFBPA), a new probability assignment based on PHFS, along with the combination rule are introduced. Moreover, we designed two methods for generating PHFBPAs using distance and the multi-classifier approach, respectively. In addition, to address the conflicts and uncertainty in the evidence, we employ an entropy measure and the Jousselme distance of PHFBPA to modify the evidence before fusion. Based on the PHFET, we develop a model for evaluating the degree of satisfaction of the SCS capability demand. Furthermore, the proposed method is applied to the classification experiments and an SCS capability demand evaluation experiment, demonstrating the feasibility and effectiveness of the PHFET approach.

The main contributions of this work are summarized as follows:

- We introduce the PHFBPA, which effectively represents information that is difficult to describe with exact values. The PHFBPA incorporates both membership degrees and probabilities, allowing for more ambiguous information representation. When the PHFBPA element is a single value, it degenerates to the basic probability assignment (BPA).
- The combination rule for PHFBPA based on the operators of PHFS and the decision-making strategy to make the final decision are proposed. A numerical example is provided to illustrate the feasibility of the combination rule.
- We develop two methods for generating PHFBPAs. The first method is based on the difference between actual and expected values, with the probability distribution of the element in the PHFE being determined by experts through system analysis. The second method is multi-classifier-based, where membership degrees and probabilities are obtained through training on a dataset.
- Discounting factors are designed to modify the PHFBPA. An entropy measure of PHFBPA is proposed as the credibility discounting factor, and its axiomatic properties are proven. The Jousselme distance of PHFBPAs is used as the reliability discounting factor to measure conflict between evidence.
- We compare the PHFET method with several machine learning algorithms for the classification of some UCI data sets, and the results demonstrate the effectiveness of the PHFET method.
- A model for evaluating SCS capability demand satisfaction degree based on PHFET is provided, which involves establishing a capability indicator system through task decomposition and fusing data from different indicators using the PHFET method. Furthermore, we simulate a representative case digitally to analyze the stability of PHFET and compare it with some traditional methods, highlighting the robustness and superiority of the PHFET method.

The remainder of the paper is structured as follows. The relevant knowledge of evidence theory, PHFS, and Jousselme distance is introduced in Section 2. In Section 3, the PHFBPA, combination rule and generation methods of PHFBPA, and discounting factors are proposed. Section 4 designs the process to evaluate the capability demand satisfaction degree of SCS. In Section 5, the PHFET is applied to target recognition and an SCS capability demand satisfaction degree evaluation. Section 6 summarizes this paper.

## 2. Preliminaries

This section briefly introduces some basic definitions of DST, PHFS, and Jousselme distance.

### 2.1. Dempster–Shafer Theory

DST is a generalization of probability theory that is useful for processing uncertain and ambiguous information. The following are the basic concepts of DST.

**Definition** **1**  (Frame of discernment)**.** *The Frame of Discernment (FOD) is a set of mutually exclusive and exhaustive hypotheses or propositions about the state of the world. It is defined as [7]:*

(1) $$\Theta =\{\theta_{1},\theta_{2},\cdots ,\theta_{n}\},$$

*where $\theta_{i}$ is an element in the frame and n is the number of hypotheses in the frame.*

**Definition** **2**  (Power set)**.** *The power set of* Θ *is denoted as follows:*

(2) $$2^{\Theta }=\left\{\emptyset ,\left\{\theta_{1}\right\},\left\{\theta_{2}\right\},\cdots ,\left\{\theta_{N}\right\},\left\{\theta_{1}\cup \theta_{2}\right\},\left\{\theta_{1}\cup \theta_{3}\right\},\cdots ,\Theta \right\}.$$

**Definition** **3**  (Basic probability assignment)**.** *Based on the power set* Θ*, the BPA, which is also called mass function, is defined as*

(3) $$m:2^{\Theta }\to \left[0,1\right],$$

*which satisfies the following conditions:*

(4) m(∅)=0,

(5) $$\sum_{A\subseteq 2^{\Theta }}m\left(A\right)=1.$$

*When m(A)>0 , A is called a focal element, and m(A) indicates the degree of evidence supporting the proposition A.*

**Definition** **4**  (Combination rule of Dempster)**.** *Let $m_{1}\left(\cdot \right)$ , $m_{2}\left(\cdot \right)$ be two BPA on* Θ*, and the combination rule of Dempster is defined as*

(6) $$m\left(A\right)=\frac{\sum_{B\cap C=A}m_{1}\left(B\right)m_{2}\left(C\right)}{1-k},$$

*with $k=\sum_{B\cap C=\emptyset }m_{1}\left(B\right)m_{2}\left(C\right)$ .*

**Definition** **5.**  *Assume m(A) to be a BPA over* Θ*. Let α∈[0,1] be the discounting factor, and the discounted BPA is defined as follows [7]:*

(7) $${\hat{m}}_{j}\left(A\right)=\left\{\begin{array}{cc} \alpha m(A)+1-\alpha , & A=\Theta \\ \alpha m(A), & A\subset \Theta ,A\neq \Theta \end{array}\right..$$

### 2.2. Probabilistic Hesitant Fuzzy Set Theory

**Definition** **6.**  *Let X be a fixed set; an HFS on X is in terms of a function that when applied to X returns a subset of [0,1] , which can be represented as the following:*

(8) H={〈x,h(x)〉∣x∈X},

*where h(x) is a set of values in [0,1] , denoting the possible membership degrees of the element x∈X to the set H. h(x) is a hesitant fuzzy element (HFE).*

**Definition** **7.**  *Let X be a reference set with finite elements; a PHFS on X is defined as:*

(9) $$H_{p}=\left\{\langle x,h_{p}\left(x\right)\rangle |x\in X\right\}.$$

*The PHFE h(x) can be described as:*

(10) $$h_{p}\left(x\right)=\left\{\gamma_{i}\left(p_{i}\right)|i=1,2,\cdots ,n\right\},$$

*where $\gamma_{i}\in \left[0,1\right]$ denotes the possible membership degrees of the element x∈X to the set $H_{p}$ , $p_{i}\in \left[0,1\right]$ is the associated occurring probability, and $\sum_{i=1}^{n}p_{i}=1$ , n is the cardinality of $h_{p}\left(x\right)$ .*

**Definition** **8.**  *The score function of the PHFE $h_{p}=\left\{\gamma_{i}\left(p_{i}\right)|i=1,2,\cdots ,n\right\}$ is defined as:*

(11) $$score\left(h_{p}\right)=\sum_{i=1}^{n}\gamma_{i}p_{i}.$$

**Definition** **9**  ([30])**.** *Given PHFEs: $h_{p1}$ and $h_{p2}$ , the multiplication operator is presented as follows:*

(12) $$\begin{array}{c} h_{p1}⊗h_{p2}=\underset{\gamma_{1}\in h_{p1},\gamma_{2}\in h_{p2}}{⋃}\left\{\left[\gamma_{1}\gamma_{2}\right]\left(p_{1}p_{2}\right)\right\} \end{array}.$$

### 2.3. Jousselme Distance

**Definition** **10**  ([34])**.** *The Jousselme distance, denoted as $d_{J}$ , is defined as follows:*

(13) $$d_{J}\left(m_{1},m_{2}\right)=\sqrt{\left.\frac{1}{2}{\left(m_{1}-m_{2}\right)}^{T}\underline{\underline{D}}(m_{1}-m_{2}\right)},$$

*where $\underline{\underline{D}}$ is a $2^{n}\times 2^{n}$ dimensional matrix, n is the number of mutually exclusive and exhaustive hypotheses, and*

(14) $$\underline{\underline{D}}\left(A,B\right)=\frac{\left|A\cap B\right|}{\left|A\cup B\right|},$$

*where |A∪B| denotes the cardinality of the subset of the union A and B. $\left|A\cap B\right|$ measures the number of common objects between elements A and B. It is easy to see that $\underline{\underline{D}}\left(A,B\right)\in \left[0,1\right]$ , and the larger $\underline{\underline{D}}\left(A,B\right)$ is, the more similar the focal elements A and B are.*

## 3. Probabilistic Hesitant Fuzzy Evidence Theory

This section introduces PHFET, including a new BPA called PHFBPA, combination rule, decision strategy, generation methods of PHFBPA, and discounting factors.

### 3.1. The Concept of the Probabilistic Hesitant Fuzzy Evidence Theory

First, referring to the definitions of PHFS, the mathematical expression and definition of the PHFBPA for focal elements are as follows:

**Definition** **11.**  *The PHFBPA of* Θ *on the power set space $2^{\Theta }$ is defined as follows:*

(15) $$m_{P}=\left\{\langle \theta ,h_{p}\left(\theta \right)\rangle |\theta \in 2^{\Theta }\right\},$$

*where $h_{p}\left(\theta \right)$ is a PHFE, $h_{p}\left(\theta \right)=\left\{\gamma_{i}\left(p_{i}\right)|i=1,2,\cdots ,n\right\}$ and $\gamma_{i}\in \left[0,1\right]$ represent the set of probability assignments of focal elements $\theta \in 2^{\Theta }$ , $p_{i}\in \left[0,1\right]$ is the associated occurring probability, and $\sum_{i=1}^{n}p_{i}=1$ , n is the cardinality of $h_{p}\left(\theta \right)$ .*

The PHFBPA embodies the hesitation between multiple quantitative single values and the importance of each expert opinion. It can be seen that if $h_{p}\left(\theta \right)$ contains only one unique value, the PHFBPA of the focal element degenerates into the classical single-value probability assignment, so that the PHFBPA of the focal element can be regarded as a generalized BPA.

The summation operation of PHFBPA is defined as the accumulation of the probability assignments and the probabilities, respectively:

(16) $$m_{P1}\left(\theta_{1}\right)+m_{P2}\left(\theta_{2}\right)=\{\left[\gamma_{1}^{\sigma (i)}+\gamma_{2}^{\sigma (i)}\right]\left(p_{1}^{\sigma (i)}+p_{2}^{\sigma (i)}\right){\}}_{i=1}^{q},$$

where $\gamma_{1}^{\sigma (i)}$ and $\gamma_{2}^{\sigma (i)}$ are the *i*-th largest values in $m_{P1}\left(\theta_{1}\right)$ and $m_{P1}\left(\theta_{2}\right)$ , and $p_{1}^{\sigma (i)}$ , $p_{2}^{\sigma (i)}$ are the associated probabilities. $q=max\{n_{1},n_{2}\}$ , in which $n_{1}$ , $n_{2}$ are the cardinality of $m_{P1}\left(\theta_{1}\right)$ , $m_{P1}\left(\theta_{2}\right)$ . If $n_{1}\neq n_{2}$ , several terms γ(p) must be provided based on the conservative criterion or the optimistic criterion [35] with the probability 0.

Based on the multiplication operator Equation (12) of PHFS, PHFBPA operators are also given to deal with focal elements:

(17) $$m_{P1}\left(\theta_{1}\right)⊗m_{P2}\left(\theta_{2}\right)=\underset{\gamma_{1}\in m_{P1}\left(\theta_{1}\right),\gamma_{2}\in m_{P2}\left(\theta_{2}\right)}{⋃}\left\{\left[\gamma_{1}\gamma_{2}\right]\left(p_{1}p_{2}\right)\right\}.$$

**Definition** **12.**  *Let $m_{P1}\left(\cdot \right)$ , $m_{P2}\left(\cdot \right)$ be two sources of evidence on* Θ*, and A,B,C are subsets of $2^{\Theta }$ . The combination rule of PHFET is defined as:*

(18) $$\forall A\in 2^{\Theta },\;m_{PM}\left(A\right)=\sum_{B,C\in 2^{\Theta },B\cap C=A}m_{P1}\left(B\right)⊗m_{P2}\left(C\right).$$

To make a final decision according to PHFBPAs, we use the score function Equation (11) to evaluate the probability of the focal element. The higher the score value, the higher the degree of trust in the proposition. Thus, the maximum support rule is adopted as the strategy of decision-making. Therefore, the maximum modulus of PHFBPA will be selected as the decision, denoted as:

(19) $$D=arg\;\underset{\theta \in D^{\Theta }}{max}\;score\left(m_{p}\left(\theta \right)\right).$$

**Example** **1.**  *Suppose that there are two PHFBPAs on the FOD $\Theta =\{\theta_{1},\theta_{2}\}$ ; they are described as follows:*

(20) $$\begin{array}{cc} m_{P1} & =\{m_{P1}\left(\theta_{1}\right),m_{P1}\left(\theta_{2}\right),m_{P1}\left(\theta_{1}\cup \theta_{2}\right)\}\\ \; & =\{\{0.6(0.8),0.5(0.2)\},\{0.2(0.5),0.1(0.5)\},\{0.3(0.7),0.2(0.3)\}\}, \end{array}$$

(21) $$\begin{array}{cc} m_{P2} & =\{m_{P2}\left(\theta_{1}\right),m_{P2}\left(\theta_{2}\right),m_{P2}\left(\theta_{1}\cup \theta_{2}\right)\}\\ \; & =\{\{0.6(0.3),0.4(0.7)\},\{0.4(0.6,0.3(0.4))\},\{0.2(0.8),0.1(0.2)\}\}. \end{array}$$

*Then, following the combination rule of PHFET, the fusion result is calculated as follows:*

(22) $$\begin{array}{cc} m_{P}\left(\theta_{1}\right) & =\sum \left\{\begin{array}{c} ⋃\left\{\begin{array}{c} m_{P1}\left(\theta_{1}\right)⊗m_{P2}\left(\theta_{1}\right),\\ m_{P1}\left(\theta_{1}\right)⊗m_{P2}\left(\theta_{1}\cup \theta_{2}\right),\\ m_{P2}\left(\theta_{1}\right)⊗m_{P1}\left(\theta_{1}\cup \theta_{2}\right) \end{array}\right\} \end{array}\right\}\\ & =\sum \left\{\begin{array}{c} ⋃\left\{\begin{array}{c} \{0.36(0.24),0.3(0.06),0.24(0.56),0.2(0.14)\},\\ \{0.12(0.64),0.1(0.16),0.06(0.16),0.05(0.04)\},\\ \left\{0.18(0.21),0.12(0.49),0.12(0.09),0.08(0.21)\right\} \end{array}\right\} \end{array}\right\}\\ & =\{0.66(1.09),0.52(0.71),0.42(0.81),0.33(0.39)\}, \end{array}$$

(23) $$\begin{array}{cc} m_{P}\left(\theta_{2}\right) & =\sum \left\{\begin{array}{c} ⋃\left\{\begin{array}{c} m_{P1}\left(\theta_{2}\right)⊗m_{P2}\left(\theta_{2}\right),\\ m_{P1}\left(\theta_{2}\right)⊗m_{P2}\left(\theta_{1}\cup \theta_{2}\right),\\ m_{P2}\left(\theta_{2}\right)⊗m_{P1}\left(\theta_{1}\cup \theta_{2}\right) \end{array}\right\} \end{array}\right\}\\ & =\sum \left\{\begin{array}{c} ⋃\left\{\begin{array}{c} \{0.08(0.3),0.06(0.2),0.04(0.3),0.03(0.2)\},\\ \{0.04(0.4),0.02(0.4),0.02(0.1),0.01(0.1)\},\\ \left\{0.12(0.42),0.09(0.28),0.08(0.18),0.06(0.12)\right\} \end{array}\right\} \end{array}\right\}\\ & =\{0.24(1.12),0.17(0.88),0.14(0.58),0.1(0.42)\}, \end{array}$$

(24) $$\begin{array}{cc} m_{P}\left(\theta_{1}\cup \theta_{2}\right) & =\sum \left\{\begin{array}{c} ⋃\left\{\begin{array}{c} m_{P1}\left(\theta_{1}\cup \theta_{2}\right)⊗m_{P2}\left(\theta_{1}\cup \theta_{2}\right) \end{array}\right\} \end{array}\right\}\\ & =\{0.06(0.56),0.04(0.24),0.03(0.14),0.02(0.06)\}. \end{array}$$

*In order for $m_{P}\left(\theta_{1}\right)$ and $m_{P}\left(\theta_{2}\right)$ to satisfy the definition of PHFBPA, the probabilities should be normalized. Then, calculate the scores of each focal element as:*

(25) $$score(m_{p}\left(\theta_{1}\right))=0.66\times 0.3633+0.52\times 0.2367+0.42\times 0.27+0.33\times 0.13=0.5192,$$

(26) $$score(m_{p}\left(\theta_{2}\right))=0.24\times 0.3733+0.17\times 0.2933+0.14\times 0.1933+0.1\times 0.14=0.1805,$$

(27) $$score(m_{p}\left(\theta_{1}\cup \theta_{2}\right))=0.06\times 0.56+0.04\times 0.24+0.03\times 0.14+0.02\times 0.06=0.0486.$$

*
$score(m_{p}\left(\theta_{1}\right))$ has the largest value; thus, the final decision is $\theta_{1}$ .*

Based on Example 1, it can be observed that after the aggregation operation, the resulting PHFBPAs should be accumulated to obtain the final PHFBPAs of the fused focal elements. In the traditional belief function framework, an overall belief mass of 1 is assigned to represent the overall belief, which is then distributed among different focal elements. However, in the case of PHFET, it requires that the score of the sum of the PHFBPAs for each focal element derived from the same source of evidence should be equal to 1 $score(\sum_{A\subseteq 2^{\Theta }}m\left(B\right))=1$ . If this condition is not met, the data must be normalized.

Let any subset of Θ be *A*; the sum of the basic belief corresponding to all subsets of *A* is referred to as the belief function, defined as:

(28) $$Bel\left(A\right)=score(\sum_{B\subseteq A}m_{P}\left(B\right)).$$

The plausibility function Pl(A) represents the non-false trust of *A* and is defined as:

(29) $$Pl\left(A\right)=score(\sum_{B\cap A\neq \phi }m_{P}\left(B\right))=1-Bel(\bar{A}).$$

### 3.2. Generation Methods of Probabilistic Hesitant Fuzzy Basic Probability Assignment

The quality of BPAs significantly impacts the result of evidence fusion and the final decision. Therefore, BPA generation is a crucial aspect of evidence theory. There are two commonly employed approaches for BPA generation. One approach involves subjective input from experts or decision-makers, drawing upon their extensive experience and knowledge. The other approach revolves around modeling the collected data to derive BPAs.

In this section, two methods for generating PHFBPAs are designed. The first method is based on distance and incorporates experts’ analysis and insights. The second method relies on a multi-classifier approach and data.

Assume there are *N* classes on FOD Θ ; *N* is the cardinality of $2^{\Theta }$ , and each class has *k* attributes, denoted as $a_{i1},a_{i2},\cdots ,a_{ik}$ .

#### 3.2.1. Distance-Based Generation Method

For practical systems, obtaining sufficient, reliable training data can be challenging. To address this issue, we propose a method for generating PHFBPAs based on the distance between sample data and ideal values derived from system analysis and experts’ opinions. The steps are as follows:

- Step 1: Define the ideal values for each class. The ideal value of class *i* is denoted as $C_{i}=\left(c_{i1},c_{i2},\cdots ,c_{ik}\right)$ , where $c_{ik}$ is the ideal value of attribute $a_{ik}$ . These values can be determined based on expert analysis of the system or through the use of clustering algorithms such as KMeans. For test sample $S=(s_{1},s_{2},\cdots ,s_{k})$ , if $s_{k}$ is equal to $C_{ik}$ , then $s_{k}$ is considered to belong to class *i*.
- Step 2: Calculate the distance between the actual and expected values. To generate the probability assignment for test sample *S*, we employ the Euclidean distance metric to quantify the dissimilarity. The Euclidean distance between *S* and each center $C_{i}$ is computed as follows:
(30) $$d_{ik}=|s_{k}-c_{ik}|.$$
- Step 3: Generate BPAs. The proximity of $s_{k}$ to a center determines the likelihood of $s_{k}$ belonging to that class. As $s_{k}$ moves further away from a center, its likelihood of belonging to that class diminishes. Therefore, the following formula is given to calculate the BPAs of the *k*th attribute:
(31) $$\gamma \left(\theta_{i}\right)=\frac{\alpha e^{\frac{-d_{ik}^{2}}{2}}}{\sum_{i=1}^{N}\alpha e^{\frac{-d_{ik}^{2}}{2}}},$$
  where α>0 is an adjustment parameter representing the impact of the distance.
- Step 4: Add the corresponding probabilities to the BPAs. The probabilities $p\left(\theta_{i}\right)\in \left(0,1\right]$ are determined by experts, and they are added to the BPAs to obtain the PHFE containing a single value $h_{p}\left(\theta_{i}\right)=\left\{\gamma \left(\theta_{i}\right)\left(p\left(\theta_{i}\right)\right)\right\}$ .
- Step 5: Select different criteria and repeat Steps 1–4. Experts may have difficulty determining the ideal value for a specific focal element due to uncertainty or variability in evaluation criteria. Thus, *n* ideal values $C_{i}^{1}\cdots C_{i}^{n}$ are determined based on different evaluation criteria for class *i*. By performing the aforementioned Steps 1–4 *n* times, we can obtain multiple elements $\gamma \left(\theta_{i}\right)\left(p\left(\theta_{i}\right)\right)$ , which collectively form the PHFBPA of attribute *k* for class *i*:
(32) $$m_{P}\left(\theta_{i}\right)=\left\{\gamma_{j}\left(\theta_{i}\right)\left(p_{j}\left(\theta_{i}\right)\right)|j=1,2,\cdots ,n\right\}.$$

#### 3.2.2. Multi-Classifier-Based Generation Method

Sometimes, obtaining accurate occurrence probabilities for uncertain elements in PHFE through the subjective judgment of experts can be challenging. To address this, we designed a method to generate PHFBPAs based on multiple classifiers. The specific steps are as follows:

- Step 1: Divide the original data set into a training set and test set.
- Step 2: Construct multiple classifiers. Utilize the training data to create *n* distinct classifiers for each piece of evidence. These classifiers are denoted as classifier 1,2,⋯,j,⋯,n . The output of each classifier should consist of sets of real numbers in [0,1] , representing the degree to which a sample belongs to different classes. The classifiers should be capable of providing the probability of each class to which a sample belongs, denoted as $y=\{y\left(\theta_{1}\right),\cdots ,y\left(\theta_{i}\right)\}$ . The accuracy of a classifier, represented as Acc , measures the proportion of correctly classified samples. It can be calculated using the following formula:
(33) $$Acc=\frac{1}{N}\sum_{i=1}^{N}\frac{TP_{i}+TN_{i}}{TP_{i}+TN_{i}+FP_{i}+FN_{i}},$$
  where *i* represents class index and *N* is the total number of classes. TP , TN , FP , and FN denote True Positives, True Negatives, False Positives, and False Negatives, respectively.
- Step 3: Generate PHFBPAs. The test set is inputted into the trained classifiers to obtain the output of each classifier. The corresponding probability based on the accuracy of each classifier is calculated as follows:
(34) $$p_{j}=\frac{Acc_{j}}{\sum_{j=1}^{n}Acc}.$$
  Subsequently, the PHFBPAs can be obtained as shown below:
(35) $$m_{P}\left(\theta \left(i\right)\right)=\left\{y_{j}\left(\theta_{i}\right)\left(p_{j}\right)|j=1,2,\cdots ,n\right\}.$$

### 3.3. Discounting Factors

Uncertainty and conflicts in evidence can result in inaccurate fusion results, thus limiting the practical application of evidence theory. Given that uncertainty and conflicts are typically caused by unreliable sources before the fusion process, we have adopted a discounting approach to estimate the reliability of evidence bodies and handle them before the fusion step. The mass function is then modified, considering both the uncertainty and distance between bodies of evidence.

#### 3.3.1. Uncertainty Measurement

According to information theory, the information quantity is proportional to its uncertainty. Conversely, evidence with lower information entropy provides more information and fosters greater confidence. As the information entropy of evidence increases, it delivers less information, involves more uncertainty, and inspires less confidence. In DST, entropy is a measure of uncertainty and disorder and has been utilized in the uncertainty measurement represented by BPA [36,37]. To measure the ambiguity and uncertainty of PHFBPA, we extend the entropy measure introduced by [38] for HFS to an entropy measure for PHFS.

In proceeding with the axiomatic definition of entropy measures for PHFBPAs, denote $A=\{\langle \theta ,h_{p}\rangle |\theta \in 2^{\Theta }\}$ as $A=\{\bar{h_{p}}\}$ , where $h_{p}$ represents a fixed PHFE.

**Definition** **13.**  *Let $m_{P}$ be a PHFBPA defined on FOD* Θ*, and an entropy measure is represented as follows:*

(36) $$\begin{array}{cc} E_{p}\left(m_{P}\right) & =\frac{1}{|m_{P}|}\sum_{i=1}^{|m_{P}|}\left(1-2\sum_{j=1}^{n}p_{j}\left(\theta_{i}\right)|\gamma_{j}\left(\theta_{i}\right)-0.5|\right)\\ \; & =1-\frac{2}{|m_{P}|}\sum_{i=1}^{|m_{P}|}\left(\sum_{j=1}^{n}p_{j}\left(\theta_{i}\right)|\gamma_{j}\left(\theta_{i}\right)-0.5|\right), \end{array}$$

*where $|m_{P}|$ is the cardinality of $m_{P}$ .*

**Proposition** **1.**  *Let A,B be two PHFBPAs; $A^{C}=\left\{\langle \theta ,h_{A^{C}}\left(\theta \right)\rangle |\theta \in 2^{\Theta }\right\}$ is the complement PHFBPA of A, where $h_{A^{C}}\left(\theta \right)=\left\{\left(1-\gamma_{j}\right)\left(p_{j}\right)|j=1,2,\cdots ,n\right\}$ . The probabilistic hesitant fuzzy entropy of the PHFBPA defined in Definition 13 has several properties as follows:*

*1.*  *
$0\leq E_{p}\left(A\right)\leq 1$ ;*
*2.*  *
$E_{P}\left(A\right)=0$ iff $A=\{\bar{0(p),1(1-p)}\}$ ;*
*3.*  *
$E_{P}\left(A\right)=1$ iff $A=\{\bar{0.5(1)}\}$ ;*
*4.*  *
$E_{P}\left(A\right)=E_{P}\left(A^{C}\right)$ ;*
*5.*  *
$E_{P}\left(A\right)\leq E_{P}\left(B\right)$ , if $\gamma_{Aj}\leq \gamma_{Bj}\leq 1/2$ or $1/2\leq \gamma_{Bj}\leq \gamma_{Aj}$ and $p_{Aj}=p_{Bj}$ .*

**Proof.** 

1. Since $0\leq \gamma (\theta_{i})\leq 1$ and $0\leq p(\theta_{i})\leq 1$ , then $0\leq |\gamma (\theta_{i})-0.5|\leq 0.5$ , and $0\leq p\left(\theta_{i}\right)|\gamma \left(\theta_{i}\right)-0.5|\leq 0.5$ . From $\sum_{j=1}^{n}p_{j}=1$ , we know $0\leq 2\sum_{j=1}^{n}p_{j}\left(\theta_{i}\right)|\gamma_{j}\left(\theta_{i}\right)-0.5|\leq 1$ . Then, $0\leq 1-2\sum_{j=1}^{n}p_{j}\left(\theta_{i}\right)|\gamma_{j}\left(\theta_{i}\right)-0.5|\leq 1$ , yielding $0\leq \frac{1}{\left|A\right|}\sum_{\left|A\right|}\left(1-2\sum_{j=1}^{n}p_{j}\left(\theta_{i}\right)|\gamma_{j}\left(\theta_{i}\right)-0.5|\right)\leq 1$ ; thus, $0\leq E_{p}\left(A\right)\leq 1$ .
2.
$E_{P}\left(A\right)=0$ iff $1-2\sum_{j=1}^{n}p_{j}\left(\theta_{i}\right)|\gamma_{j}\left(\theta_{i}\right)-0.5|=0$ iff $\sum_{j=1}^{n}p_{j}\left(\theta_{i}\right)|2\gamma_{j}\left(\theta_{i}\right)-1|=1$ , then $\gamma (\theta_{i})=0$ or $\gamma (\theta_{i})=1$ , A={0(p),1(1−p)} .
3.
$E_{P}\left(A\right)=1$ iff $1-2\sum_{j=1}^{n}p_{j}\left(\theta_{i}\right)|\gamma_{j}\left(\theta_{i}\right)-0.5|=1$ iff $\sum_{j=1}^{n}p_{j}\left(\theta_{i}\right)|2\gamma_{j}\left(\theta_{i}\right)-1|=0$ iff $\gamma (\theta_{i})=0.5$ .
4. Since $A^{C}=⋃\left\{1-\gamma_{j}\left(\theta_{i}\right)\left(p_{j}\left(\theta_{i}\right)\right)\right\}$ , then $E_{p}\left(A\right)=1-\frac{2}{|m_{p}|}\sum_{i=1}^{|m_{p}|}\left(\sum_{j=1}^{n}p\left(\theta_{i}\right)|1-\gamma \left(\theta_{i}\right)-0.5|\right)=E_{p}\left(A^{C}\right)$ .
5. If $\gamma_{Aj}\leq \gamma_{Bj}\leq 1/2$ or $1/2\leq \gamma_{Bj}\leq \gamma_{Aj}$ and $p_{Aj}=p_{Bj}$ , then $|\gamma_{Aj}\left(\theta_{i}\right)-0.5|\geq |\gamma_{Bj}\left(\theta_{i}\right)-0.5|$ . Hence, $1-2\sum_{j=1}^{n}p_{Aj}\left(\theta_{i}\right)|\gamma_{Aj}\left(\theta_{i}\right)-0.5|\leq 1-2\sum_{j=1}^{n}p_{Bj}\left(\theta_{i}\right)|\gamma_{Bj}\left(\theta_{i}\right)-0.5|$ , which implies $E_{P}\left(A\right)\leq E_{P}\left(B\right)$ .

□

The credibility discounting factor can be obtained as:

(37) $$\omega_{i}=\left\{\begin{array}{cc} \frac{1/E_{p}\left(m_{i}\right)}{\sum_{i=1}^{k}1/E_{p}\left(m_{i}\right)}, & E_{p}\left(m_{i}\right)\neq 0\\ 1/k, & E_{p}\left(m_{i}\right)=0 \end{array}\right.,$$

in which *k* is the number of bodies of evidence.

#### 3.3.2. Conflict Measurement

The Jousselme evidence distance is an effective approach to estimate the presence of conflicts between evidence. Therefore, we establish the reliability of evidence based on the Jousselme evidence distance. According to Definition 10, the Jousselme distance of PHFBPA is given as follows:

**Definition** **14.**  *Let $m_{P1}$ and $m_{P2}$ be two PHFBPAs defined on the same FOD* Θ*; A and B are any focal elements of $m_{P1}$ and $m_{P2}$ . The Jousselme distance, denoted as $d_{p}$ , is defined as*

(38) $$d_{p}\left(m_{P1},m_{P2}\right)=\sqrt{\left.\frac{1}{2}{\left(m_{P1}-m_{P2}\right)}^{T}\underline{\underline{D}}(m_{P1}-m_{P2}\right)},$$

*in which $\underline{\underline{D}}$ is an N×N dimensional matrix, N is the cardinality of $2^{\Theta }$ ,*

(39) $$m_{P1}-m_{P2}=\left[\begin{array}{c} h_{p1}\left(\theta_{1}\right)⊖h_{p2}\left(\theta_{1}\right)\\ h_{p1}\left(\theta_{2}\right)⊖h_{p2}\left(\theta_{2}\right)\\ \vdots \\ h_{p1}\left(\theta_{N}\right)⊖h_{p2}\left(\theta_{N}\right) \end{array}\right],$$

*and for $\theta_{i}\in 2^{\Theta }$ :*

(40) $$h_{p1}\left(\theta_{i}\right)⊖h_{p2}\left(\theta_{i}\right)=\sum_{j=1}^{n}\left(p_{j}^{1}\left(\theta_{i}\right)\gamma_{j}^{1}\left(\theta_{i}\right)-p_{j}^{2}\left(\theta_{i}\right)\gamma_{j}^{2}\left(\theta_{i}\right)\right),$$

*where n is the cardinality of $h_{p}\left(\theta_{i}\right)$ .*

The degree of similarity can be defined as

(41) $$Sim\left(m_{P1},m_{P2}\right)=1-d_{P}\left(m_{P1},m_{P2}\right).$$

The reliability discounting factor can be quantified by the support degree, which is defined as below:

(42) $$\upsilon_{i}=\frac{\sum_{j=1,j\neq i}^{k}s\left(m_{ij}\right)}{\sum_{j=1}^{k}\sum_{j=1,j\neq i}^{k}s\left(m_{i}\right)},$$

where *k* is the number of bodies of evidence.

Before combining the evidence, it is essential to adjust the PHFBPAs with the credibility discounting factor and the reliability discounting factor. However, in practice, inconsistency in the status of different information sources leads to different levels of importance for different evidence. Consequently, an importance weight is assigned to every body of evidence to accurately reflect its relative importance in the evaluation process. Let the importance weight be δ , and $\sum_{i=1}^{k}\delta_{i}=1$ . Then, the normalized weight assigned to each body of evidence is as follows:

(43) $$\alpha_{i}=\delta_{i}\frac{\omega_{i}\upsilon_{i}}{\sum_{i=1}^{k}\omega_{i}\upsilon_{i}}.$$

According to Definition 5 and the weight of each body of evidence, the discounted PHFBPAs are as follows:

(44) $$m_{P}\left(\theta \right)=\sum_{i=1}^{k}\alpha_{i}m_{Pi}\left(\theta \right).$$

Then, use the combination rule in Definition 12 to fuse the modified evidence for k−1 times to obtain the final result.

## 4. Capability Evaluation of a Satellite Communication System

Capability refers to the function, performance, and efficiency that a system must possess to successfully accomplish a specific mission. In this section, an evaluation model based on evidence theory is established to analyze whether the current capability of the SCS aligns with the requirements of the mission task.

By decomposing the mission task and conducting system analysis, we identify the specific demands associated with the mission. Subsequently, we construct a comprehensive capability indicator system that encompasses these demands. To measure the satisfaction level of the capability demands, we employ the PHFET to fuse data from different indicators.

### 4.1. Capability Indicator System Construction

**Figure 1:**
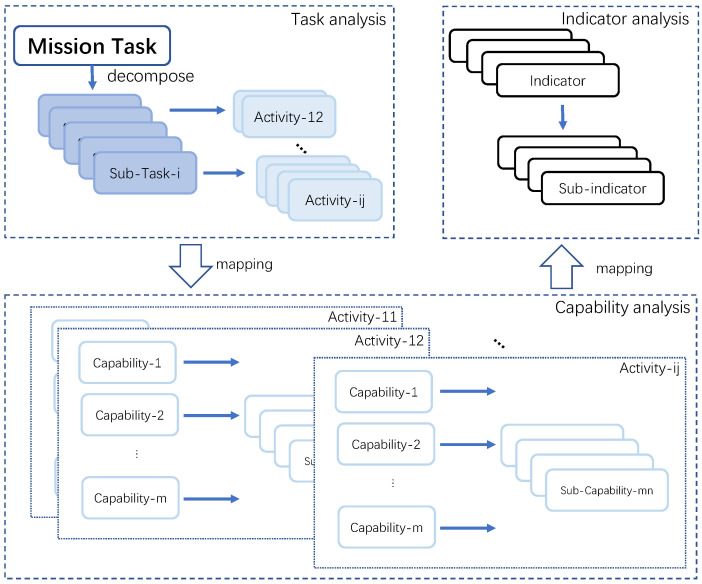
Indicator system construction process.

In order to establish a comprehensive evaluation model for the capability demand satisfaction of SCS, it is necessary to decompose the demands into specific capabilities that can be assessed. In this regard, a set of capability indicators must be established to assess the level of performance for each capability. These indicators should be measurable, reflect the key attributes of each capability, and be structured in a hierarchical system. Therefore, a thorough analysis of the demands and capabilities is required to establish an evaluation model that can effectively guide the development of the SCS. The process of constructing the indicator system is illustrated in Figure 1.

- Step 1: Task analysis. The initial step involves the decomposition of mission tasks to identify the capabilities that are necessary to support these tasks. Given that mission tasks are diverse in nature, the capabilities required to accomplish them are also varied. Thus, by breaking down the core mission task *T*, we obtain independent and unique sub-tasks at different levels, denoted as
(45) $$T=\{T_{1},T_{2},\cdots ,T_{i}\}.$$
  Secondly, refine tasks into activity options. The unit-level activities *A* describe the specific behavior required to complete the tasks, and their corresponding relationships with capabilities are relatively stable, allowing for mapping with capability indicators. We gradually decompose the tasks until we reach activities that correspond to capability indicators
(46) $$T_{i}=\left\{A_{i}^{1},A_{i}^{2},\cdots ,A_{i}^{j}\right\}.$$
  It is worth noting that lower-level tasks can simultaneously support multiple upper-level tasks. To enhance the accuracy of our task description, we aggregate the final level of sub-tasks and activities, eliminating any duplication or redundancy.
- Step 2: Capability analysis. The specific execution of an activity requires certain capabilities, creating a one-to-one or one-to-many mapping relationship between activities and capabilities, denoted as follows:
(47) $$A_{i}^{j}=\left\{Ca_{1},Ca_{2},\cdots ,Ca_{m}\right\}.$$
  Capabilities are further broken down into multiple sub-capabilities until they arrive at measurable capability indicators.
(48) $$Ca_{m}=\left\{ca_{m}^{1},ca_{m}^{2},\cdots ,ca_{m}^{n}\right\},$$
  representing *n* sub-capabilities under capability $Ca_{m}$ .
- Step 3: Indicator analysis. A capability is defined by one or more capability indicators $ca_{m}^{n}=\left\{I_{1},I_{2},\cdots \right\}$ , which are measurable capability attributes. We decompose the capabilities to obtain sub-capabilities, and this iterative decomposition process continues until we reach a set of basic measurable, operable, and understandable attributes, that is, technical and tactical indicators of the system.

**Figure 2:**
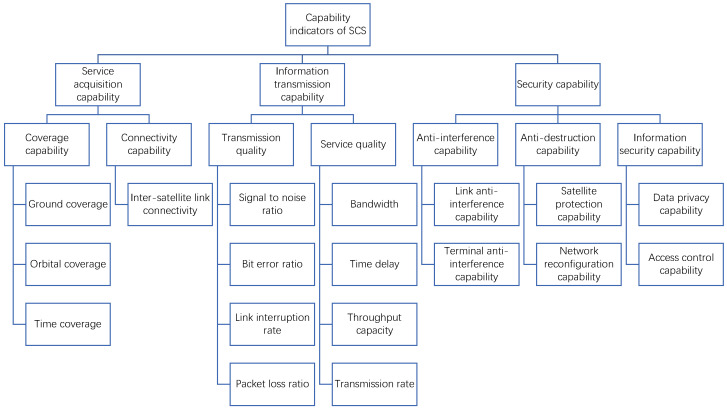
Capability indicator system of SCS.

The SCS primarily performs information transmission tasks. The degree of task completion varies depending on its information transmission capability, information security and protection capability, and other factors. Through the process outlined above, we have constructed a general evaluation indicator system for the SCS, consisting of three main capability indicators: service acquisition capability, information transmission capability, and security capability. Moreover, each of the main indicators was decomposed into multiple quantifiable individual indicators. As a result, we have obtained a hierarchical capability indicator system for the SCS, as shown in Figure 2.

### 4.2. Capability Demand Satisfaction Degree Evaluation

**Figure 3:**
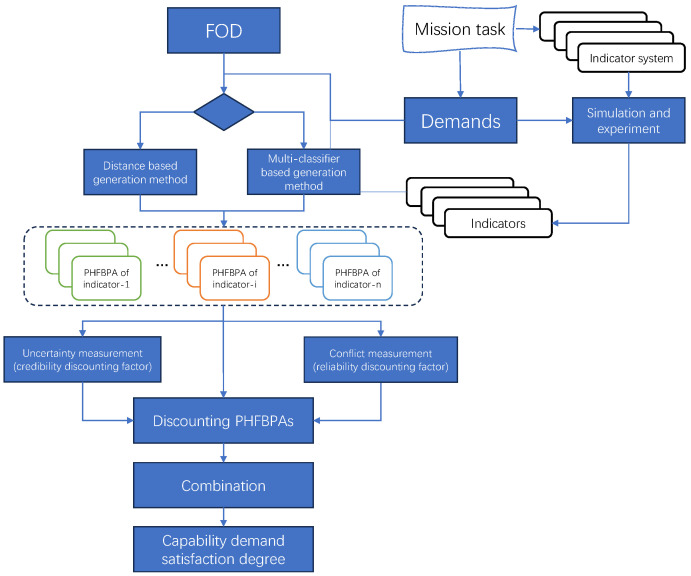
Capability demand satisfaction evaluation process.

After obtaining the capability indicators for a specific mission, we employ PHFET to obtain the PHFBPA of each indicator and then combine the results in order to evaluate the overall capability demand satisfaction degree of the SCS. The process of the evaluation is shown in Figure 3.

To evaluate the satisfaction degree of the SCS using evidence theory, it is essential to establish the appropriate frame before combining information expressed as belief functions. Each element in the frame of discernment represents a level of satisfaction with the capabilities required for the task. For example, we define $\Theta =\{\theta_{1},\theta_{2}\}$ , where $\theta_{1}$ indicates that the SCS possesses the capacity to meet the demands, and $\theta_{2}$ suggests that the capabilities of the SCS do not meet the demands. Hence, when the degree of satisfaction is divided into *N* levels, the frame of discernment is established as follows:

(49) $$\Theta =\{\theta_{1},\theta_{2},\cdots ,\theta_{N}\},$$

where $\theta_{N}$ represents the *N*th degree of satisfaction.

Firstly, we design the simulation experiments under specific operational scenarios to obtain the experimental data of each indicator according to the mission task and indicator system. Subsequently, these experimental data are utilized to calculate the PHFBPAs of each indicator for each class of satisfaction under the established FOD. In cases where there are insufficient training data, the distance-based method proposed in Section 3.2.1 can be employed, where the ideal value of the indicator represents the desirable value that must be achieved to complete the task. Conversely, the muti-classifier based method proposed in Section 3.2.2 can be used to derive the PHFBPAs. Then, we discount the PHFBPAs by solving the uncertainty and conflicts. Moreover, the different importance of each indicator can be adjusted by adding importance weights to the PHFBPAs. Finally, combine the PHFBPA of all indicators to obtain the capability demand satisfaction degree of the SCS under a specific mission.

It is worth noting that the ideal value of the same indicator under different sub-tasks can take different values, thus constituting multiple degrees of membership values for each element in the PHFBPA. And the probability of each PHFE in the PHFBPA can also indicate the importance of sub-tasks.

## 5. Verification and Application

In this section, two types of experiments are conducted to evaluate the performance of the PHFET on classification and capability demand satisfaction degree evaluation. To achieve this, we utilize data sets obtained from the UCI machine learning repository, alongside a case simulation of SCS.

### 5.1. Verification on Classification

The applications in this study utilize several data sets sourced from the UCI machine learning database. These data sets include the Statlog (Australian Credit Approval) data set, Breast Cancer Wisconsin (Diagnostic) data set, Seeds data set, Climate Model Simulation Crashes data set (CMSC), Heart disease data set, Wine data set, and Ionosphere data set. The details are shown in Table 1.

To demonstrate the effectiveness of the proposed method in classification, several algorithms are selected. These algorithms include XGBoost, Support Vector Machine (SVM), Random Forest (RF), Neural Network (NN), and Logistic Regression (LR). For each dataset, we have chosen ten classifiers from these five methods to generate a diverse set of classifiers. The specific classifiers used are as follows:

1. XGBoost classifiers with booster options of gbtree and gblinear.
2. SVM classifiers with radial basis function kernel and linear kernel.
3. RF classifiers with criterion options of gini and entropy.
4. Multi-Layer Perceptron (MLP) classifiers with two hidden layers using either 10-10 or 20-10 nodes, and tansig activation function.
5. LR classifiers with LBFGS and Stochastic Average Gradient (SAG) solver, respectively.

To combine the classifiers, the PHFET method is used. Five types of classifiers are utilized, resulting in five bodies of evidence. Each body of evidence includes information from two classifiers. The mass function of evidence is generated using the output of two classifiers and their accuracy, according to Equations (34) and (35). In the experiments, the 5-fold cross-validation method is employed, which is a common method to test the accuracy of the classification algorithm. The mean accuracy of different classifiers based on different data sets is provided in Table 2.

As observed from Table 2, the PHFET method consistently achieves the highest accuracy across all seven data sets. These results clearly demonstrate the effectiveness of the PHFET method in merging the information and advantages of multiple classifiers, leading to improved recognition accuracy.

### 5.2. Application on Capability Demand Satisfaction Evaluation of SCS

To demonstrate the effectiveness of a practical SCS capability evaluation, a specific information assurance mission is considered. This evaluation aims to assess the SCS’s ability to meet the requirements of the mission task. The SCS consists of the walker constellation, with a total of 24 satellites, 4 orbital planes, and 6 satellites per orbital plane.

The objective of this operation is to safeguard maritime and land-based communications, which can be further divided into three tasks: command communications, reconnaissance intelligence transmission, and daily communications tasks. These activities involve issuing command orders, transmitting and receiving positioning information and weather updates, facilitating daily communications, and providing broadcasting services. By analyzing each activity individually, a list of SCS capability demands under the information assurance mission is compiled, along with corresponding capability indicators. These indicators include ground coverage, orbital coverage, time coverage, inter-satellite link connectivity, signal-to-noise ratio, bit error ratio, link interruption rate, packet loss ratio, bandwidth, time delay, transmission rate, throughput capacity, denoted as $I_{1}$ to $I_{12}$ .

The evaluation considers three concentrations within the FoD:

(50) $$V=\left\{v_{1},v_{2},v_{3}\right\},$$

where $v_{1},v_{2},v_{3}$ represent the satisfaction levels of high, medium, and low, and the boundaries of $v_{1},v_{2}$ , and $v_{2}$ are not precisely defined.

Assume that there are two potential schemes for constructing the SCS to fulfill the mission. The demand indicator values of different satisfaction levels have been provided by experts, and the indicator values of different schemes are collected from simulation. The values of the demand indicators for maritime communication are 0.9 , 0.8 , 0.8 , 0.75 , 0.7 , 0.95 , 0.95 , 0.8 , 0.9 , 1.1 , 0.9 , 0.9 times higher than that for land, respectively.

In order to integrate the different indicator values, the raw data need to be standardized and converted into normalized data with a range of [0,1] . The demand indicators of the land communication assurance mission and the indicators of two schemes are given in Table 3.

The importance of two parts of the mission under 12 indicators is shown in Table 4.

According to the distance-based generation method, the data of 12 indicators are modeled as PHFBPAs, which consist of the importance and the degree of affiliations of demand of two parts of the mission. The detailed PHFBPAs of scheme 1 are shown in Table 5.

As depicted in Table 5, an inconsistency arises between $m_{P8}$ and other evidence since $m_{P8}$ assigns more belief mass to satisfaction level $v_{2}$ compared to $v_{1}$ , which is supported by other evidence. To ensure a comprehensive synthesis, it is crucial to merge the various pieces of data. Relying solely on a single piece of evidence would be unreliable for making informed decisions. Therefore, in order to address the uncertainties and conflicts, the reliability discounting factor is determined by employing the Jousselme distance of PHFBPAs, while the credibility discounting factor is determined using the entropy measure of PHFBPAs.

According to Equation (37), the credibility discounting factors could be calculated as follows:

(51) $$\omega ={\left[0.0833\;0.0833\;0.0833\;0.0824\;0.0814\;0.0839\;0.0836\;0.0833\;0.0838\;0.0847\;0.0838\;0.0833\right]}^{T}.$$

Following Equation (42), the reliability discounting factors could be calculated as:

(52) $$\upsilon ={\left[0.0834\;0.0834\;0.0834\;0.0826\;0.0816\;0.0838\;0.0837\;0.0834\;0.0836\;0.0844\;0.0836\;0.0833\right]}^{T}.$$

The credibility discounting factors and the reliability discounting factor are integrated to form the final weight to adjust the PHFBPAs of the evidence. Applying the combination rule of PHFET Equation (18) to fuse the modified evidence 11 times and use the score function of PHFBPA to obtain the final result as: $score\left(m_{p1}\left(v_{1}\right)\right)=0.8725,score\left(m_{p1}\left(v_{2}\right)\right)=0.127,$ $score(m_{p1}\left(v_{3}\right))=0.0005$ . These scores indicate a high degree of satisfaction with the capability demand for scheme 1, suggesting that the Satellite Communication System (SCS) built according to this scheme possesses the necessary capabilities to successfully fulfill the mission.

**Figure 4:**
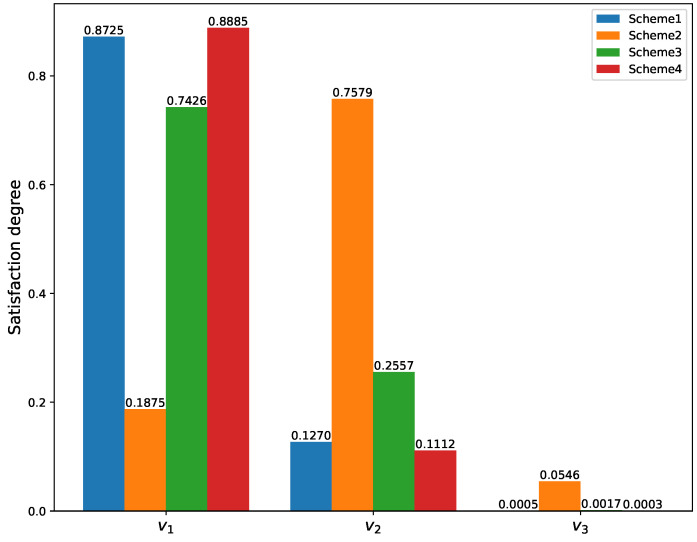
Satisfaction degrees of different schemes.

To facilitate a comparison between different schemes, assume that there are scheme 3 and scheme 4. Scheme 3 shares identical indicators with scheme 1, except for $I_{6}=0.896$ and $I_{7}=0.8267$ . On the other hand, scheme 4 has identical indicators to scheme 1, except for $I_{8}=0.335$ . The fusion result for these four schemes are illustrated in Figure 4.

Analyzing the satisfaction degrees of the four schemes reveals that scheme 1, scheme 3, and scheme 4 exhibit high levels of satisfaction, while scheme 2 demonstrates a moderate level. The discrepancies in the indicator values account for the variations in satisfaction levels among scheme 1, scheme 3, and scheme 4. Notably, scheme 3 yields the highest level of satisfaction. However, despite a notable reduction in packet loss ratio compared to scheme 1, its impact on overall improvement is minimal.

### 5.3. Discussion

To assess and validate the stability of the proposed algorithm, we conducted sensitivity analysis on the indicator weights and mission importance to examine their impact on the fusion result. We assigned different weights to the 12 indicators, denoted as δ in Equation (43), creating three distinct weight sets, as shown in Table 6.

Furthermore, in order to evaluate the effect of the basis for the possibility values in PHFBPAs on the fusion result, Table 7 presents two more importance ratings for land and maritime communication missions in addition to those in Table 4.

**Figure 5:**
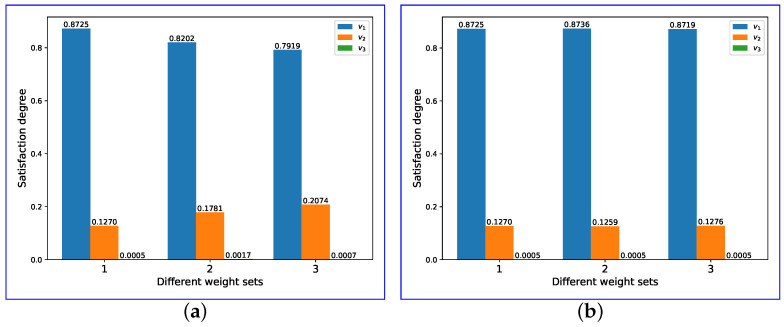
Stability analysis. (**a**) Fusion results with different indicator weights; (**b**) fusion results with different mission importance.

The experimental results, depicted in Figure 5, reveal that the priority order of satisfaction level remains consistent despite significant fluctuations in both indicator weights (Figure 5a) and mission importance (Figure 5b). These findings strongly support the stability and robustness of the introduced PHFET model under various weighting scenarios.

These results reaffirm the effectiveness and reliability of the proposed algorithm for the decision-making processes. The algorithm’s ability to maintain consistent performance across different weight configurations enhances its practical applicability. Decision-makers can confidently use this model without concerns about unpredictable or inconsistent outcomes due to variations in experts’ weights. Additionally, the stability analysis provides a solid foundation for future research and potential refinements of the algorithm.

Several traditional methods have been adopted for comparison, including Dempster’s method [39], referred to as ‘DS’; Yager’s method [40], referred to as ‘Yager’; Sun et al.’s method [41], referred to as ‘Sun’; Murphy’s method [42], referred to as ‘Murphy’; and Deng’s method [36], referred to as ‘Deng’. To verify the effectiveness of the proposed discounting factors in eliminating uncertainty and conflicting evidence, several variants of the PHFET method are utilized. The PHFET method without the discounting of evidence is denoted as ‘PHFET’, while the versions with credibility discounting factor and reliability discounting factor are denoted as ‘PHFET-1’ and ‘PHFET-2’, respectively. Furthermore, the combination of both discounting factors is denoted as ‘PHFET-12’.

**Figure 6:**
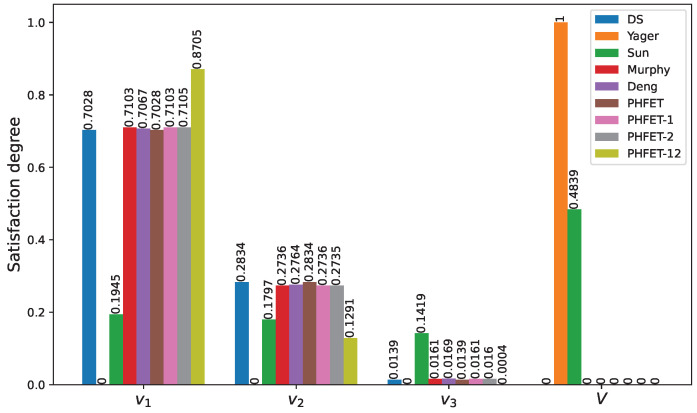
Fusion results of different methods.

According to the distance-based generation method proposed in the previous section, PHFBPAs of scheme 1 were obtained. Furthermore, to facilitate comparison with other methods, the adjustment parameter α in Equation (31) takes the values of 1 and 1, with a probability of 0.5 for each value. The final fusion results of the evidence from all indicators are depicted in Figure 6.

As can be seen from Figure 6, most of the methods allocate the largest belief mass to $v_{1}$ , indicating sufficient capabilities to carry out the mission as intended, except for Yager’s method and Sun et al.’s method. These two methods allocate most of the belief mass to an unknown space *V*, indicating that they cannot provide a specific satisfaction level. Among the methods that identify the satisfaction level as high, the discounted PHFET method performs the best, achieving the highest belief of 0.8705 and demonstrating superior convergence performance by quickly converging to 1. Additionally, compared to the PHFET method without discounting, which assigned the belief mass of $v_{1}$ to 0.7028 , and only uses one of the credibility and reliability discounting factors, which results in belief masses of 0.7103 and 0.7105 , respectively, the PHFET method with both discounting factors allocates a higher belief degree to the target concentration. Thus, the effectiveness and superiority of the uncertainty and conflict-based discounting strategy of PHFET is demonstrated.

## 6. Conclusions

In this paper, we introduce a generalized form of BPA that incorporates fuzziness and hesitancy, PHFBPA, which extends DST to the PHFET. Two novel methods based on distance and multi-classifier approaches are designed for generating PHFBPAs. Moreover, the combination rule that integrates PHFBPA with a decision-making strategy are proposed. To address the inconsistency of evidence, we employ a discounting method with an entropy measure of PHFBPA as a credibility discounting factor and the Jousselme distance of PHFBPAs as a reliability discounting factor. Furthermore, we establish and apply a PHFET model for evaluating the satisfaction degree of the SCS capability demand in a specific case study. Experimental results demonstrate the effectiveness and superiority of the PHFET method compared to various machine learning algorithms and traditional methods applied to classification tasks on different data sets. The consistent outperformance of the PHFET method highlights its enhanced capability and potential for practical applications.

In our future works, one potential direction is to refine and optimize the combination rule and decision-making strategies of PHFET in order to enhance the efficiency and accuracy of decision-making processes. Additionally, it is essential to dedicate further efforts towards improving the computational efficiency of PHFET algorithms, particularly when handling large-scale data sets, as they can become computationally intensive. Moreover, it would be worthwhile to explore the integration of PHFET with emerging techniques, such as deep learning or ensemble learning, and their application across various domains. 

## Figures and Tables

**Table 1:** Basic information of the data sets.

Data Set	Attribute Type	Instances	Attributes	Class	Subject Area
Statlog	Categorical, Integer, Real	690	14	2	Financial
Breast Cancer	Real	569	30	2	Life
Seeds	Real	210	7	3	Life
CMSC	Real	540	18	2	Physical
Heart disease	Categorical, Integer, Real	303	13	5	Life
Wine	Integer, Real	178	13	3	Physical
Ionosphere	Integer, Real	351	34	2	Physical

**Table 2:** Classification accuracy of different methods.

Data Set	XGBoost-1	XGBoost-2	SVM-1	SVM-2	RF-1	RF-2	MLP-1	MLP-2	LR-1	LR-2	PHFET
Statlog	0.8810	0.8578	0.8564	0.8534	0.8810	0.8883	0.8549	0.8549	0.8593	0.8593	0.8905
Breast cancer	0.9701	0.9649	0.9210	0.9210	0.9649	0.9649	0.9139	0.8402	0.9438	0.9139	0.9912
Seeds	0.9191	0.9571	0.9381	0.9429	0.9143	0.8952	0.9143	0.9000	0.9381	0.9381	0.9762
CMSC	0.9204	0.9204	0.9204	0.9500	0.9500	0.9556	0.9574	0.9611	0.9630	0.9630	0.9630
Heart disease	0.5710	0.5876	0.5808	0.5842	0.5841	0.5775	0.5940	0.6007	0.5875	0.5875	0.6333
Wine	0.9492	0.9552	0.6571	0.9494	0.9776	0.9775	0.6356	0.6395	0.9494	0.7135	1.0000
Ionoshpere	0.9345	0.8805	0.9402	0.8804	0.9402	0.9375	0.8719	0.8803	0.8803	0.8803	0.9486

**Table 3:** Values of capability indicators.

Indicators	Demand Value of $v_{1}$	Demand Value of $v_{2}$	Demand Value of $v_{3}$	Scheme 1	Scheme 2
$I_{1}$	0.9679	0.7364	0.6312	1	0.9772
$I_{2}$	0.8878	0.6658	0.4439	0.9903	1
$I_{3}$	1	0.9	0.7	0.9772	0.9897
$I_{4}$	1	0.5	0.2	0.7789	0.8355
$I_{5}$	0.9672	0.9188	0.8705	0.9636	1
$I_{6}$	0.7921	0.9307	1	0.8267	0.8239
$I_{7}$	0.1667	0.6667	1	0.3553	0.6927
$I_{8}$	0.3333	0.5	1	0.4517	0.8553
$I_{9}$	1	0.9333	0.8333	0.9719	0.9718
$I_{10}$	0.8	0.8571	1	0.8083	0.8040
$I_{11}$	1	0.8947	0.7895	0.9704	0.9742
$I_{12}$	1	0.5	0.3125	0.9375	0.5000

**Table 4:** Importance of mission.

Importance	$I_{1}$	$I_{2}$	$I_{3}$	$I_{4}$	$I_{5}$	$I_{6}$	$I_{7}$	$I_{8}$	$I_{9}$	$I_{10}$	$I_{11}$	$I_{12}$
Land communication	0.6	0.8	0.8	0.7	0.6	0.75	0.5	0.8	0.8	0.8	0.8	0.6
Maritime communication	0.4	0.2	0.2	0.3	0.4	0.25	0.5	0.2	0.2	0.2	0.2	0.4

**Table 5:** PHFBPAs of scheme 1.

PHFBPAs	$m_{P}\left(v_{1}\right)$	$m_{P}\left(v_{2}\right)$	$m_{P}\left(v_{3}\right)$
$m_{P1}$	{0.3794(0.6), 0.3415(0.4)}	{0.3309(0.6), 0.2978(0.4)}	{0.2898(0.6), 0.26070(0.4)}
$m_{P2}$	{0.4185(0.8), 0.3348(0.2)}	{0.3463(0.8), 0.2770(0.2)}	{0.2352(0.8), 0.18819(0.2)}
$m_{P3}$	{0.3512(0.8), 0.2809(0.2)}	{0.3474(0.8), 0.2779(0.2)}	{0.3015(0.8), 0.2412(0.2)}
$m_{P4}$	{0.3987(0.7), 0.2990(0.3)}	{0.3763(0.7), 0.2823(0.3)}	{0.2250(0.7), 0.1687(0.3)}
$m_{P5}$	{0.3357(0.6), 0.2350(0.4)}	{0.3344(0.6), 0.2341(0.4)}	{0.3299(0.6), 0.2310(0.4)}
$m_{P6}$	{0.3419(0.75),0.3248(0.25)}	{0.3354(0.75),0.3186(0.25)}	{0.3227(0.75),0.3066(0.25)}
$m_{P7}$	{0.4251(0.5), 0.4039(0.5)}	{0.3761(0.5), 0.3573(0.5)}	{0.1988(0.5), 0.1889(0.5)}
$m_{P8}$	{0.3865(0.8), 0.3092(0.2)}	{0.3956(0.8), 0.3165(0.2)}	{0.2179(0.8), 0.1743(0.2)}
$m_{P9}$	{0.3376(0.8), 0.3038(0.2)}	{0.3371(0.8), 0.3034(0.2)}	{0.3254(0.8), 0.2928(0.2)}
$m_{P10}$	{0.3419(0.8), 0.3761(0.2)}	{0.3403(0.8), 0.3744(0.2)}	{0.3177(0.8), 0.3495(0.2)}
$m_{P11}$	{0.3415(0.8), 0.3073(0.2)}	{0.3382(0.8), 0.3044(0.2)}	{0.3204(0.8), 0.2884(0.2)}
$m_{P12}$	{0.4654(0.6), 0.4189(0.4)}	{0.3199(0.6), 0.2879(0.4)}	{0.2147(0.8), 0.1933(0.4)}

**Table 6:** Weight sets of indicators.

Weight Set	$I_{1}$	$I_{2}$	$I_{3}$	$I_{4}$	$I_{5}$	$I_{6}$	$I_{7}$	$I_{8}$	$I_{9}$	$I_{10}$	$I_{11}$	$I_{12}$
1	1/12	1/12	1/12	1/12	1/12	1/12	1/12	1/12	1/12	1/12	1/12	1/12
2	0.151	0.032	0.312	0.012	0.144	0.132	0.035	0.067	0.005	0.014	0.081	0.015
3	0.017	0.051	0.081	0.036	0.123	0.092	0.094	0.225	0.068	0.097	0.106	0.01

**Table 7:** Importance sets of missions.

Importance Set	$I_{1}$	$I_{2}$	$I_{3}$	$I_{4}$	$I_{5}$	$I_{6}$	$I_{7}$	$I_{8}$	$I_{9}$	$I_{10}$	$I_{11}$	$I_{12}$
2	0.96	0.52	0.7	0.32	0.27	0.4	0.67	0.18	0.63	0.73	0.68	0.54
	0.04	0.48	0.3	0.68	0.73	0.6	0.33	0.82	0.37	0.27	0.32	0.46
3	0.05	0.06	0.79	0.98	0.96	0.24	0.19	0.26	0.49	0.82	0.25	0.39
	0.95	0.94	0.21	0.02	0.04	0.76	0.81	0.74	0.51	0.18	0.75	0.61

## Data Availability

All data generated or analyzed during this study are included in this published article.
